# Author Correction: Fast and scalable production of crosslinked polyimide aerogel fibers for ultrathin thermoregulating clothes

**DOI:** 10.1038/s41467-024-45600-9

**Published:** 2024-02-07

**Authors:** Tiantian Xue, Chenyu Zhu, Dingyi Yu, Xu Zhang, Feili Lai, Longsheng Zhang, Chao Zhang, Wei Fan, Tianxi Liu

**Affiliations:** 1 State Key Laboratory for Modification of Chemical Fibers and Polymer Materials, College of Materials Science and Engineering, Donghua University, 2999 North Renmin Road, Shanghai, 201620 China; 2 Key Laboratory of Synthetic and Biological Colloids, Ministry of Education, School of Chemical and Material Engineering, Jiangnan University, Wuxi, 214122 P. R. China; 3 State Key Laboratory of Metal Matrix Composites, School of Materials Science and Engineering, Shanghai Jiao Tong University, Shanghai, 200240 P. R. China

**Keywords:** Polymers, Synthesis and processing, Polymers

Correction to: *Nature Communications* 10.1038/s41467-023-43663-8, published online 16 December 2023

The original version of this Article contained an error in Fig. 1a in the manuscript file and Supplementary Fig. 8 in the Supplementary Information File, in which the molecular structures represented contain errors. This has been corrected in both the PDF and HTML versions of the Article and the PDF version of the Supplementary Information File.

### Supplementary information

Updated Supplementary Information

