# Supplementary material for: Fast and scalable production of crosslinked polyimide aerogel fibers for ultrathin thermoregulating clothes
Source: Nat Commun. 2023 Dec 16;14:8378. doi: 10.1038/s41467-023-43663-8 (PMC10725485; doi:10.1038/s41467-023-43663-8)
Supplement: Supplementary file 1 — Supplementary information [file 41467_2023_43663_MOESM1_ESM.pdf]

Supplementary information

Fast and Scalable Production of Crosslinked Polyimide Aerogel Fibers for Ultrathin Thermoregulating Clothes

Tiantian Xue[1], Chenyu Zhu[1], Dingyi Yu[1], Xu Zhang[2], Feili Lai[3], Longsheng Zhang[2], Chao Zhang[1], Wei Fan[1,2]*, Tianxi Liu[1,2]*

[1] State Key Laboratory for Modification of Chemical Fibers and Polymer Materials, College of Materials Science and Engineering, Donghua University, 2999 North Renmin Road, Shanghai 201620, P. R. China.

[2] Key Laboratory of Synthetic and Biological Colloids, Ministry of Education, School of Chemical and Material Engineering, Jiangnan University, Wuxi 214122, P.R. China.

[3] State Key Laboratory of Metal Matrix Composites, School of Materials Science and Engineering, Shanghai Jiao Tong University, Shanghai, 200240, P. R. China.

* Corresponding authors. E-mail address: weifan@jiangnan.edu.cn or weifan@dhu.edu.cn (W. Fan); txliu@jiangnan.edu.cn (T. X. Liu).

Table of Contents

1 Coarse-grained molecular dynamics simulations

2 Supplementary Figures and Tables

Supplementary Fig. 1 Synthetic chemical reaction of photosensitive polyimide (PPI).
Supplementary Fig. 2 Structure characterization of PI and PAA.
Supplementary Fig. 3 Molecular weight distribution curve of soluble PI.
Supplementary Fig. 4 FTIR spectra and ^{1}H NMR spectra of PPI with different HEMA grafting ratios.
Supplementary Fig. 5 Molecular weight distribution curve of PPI.
Supplementary Fig. 6 Storage stability experiments of PPI spinning solution.
Supplementary Fig. 7 Apparent viscosity as a function of shear rate for PPI solution with different HEMA grafting ratios.
Supplementary Fig. 8 Chemical structure of PPI and CPI under UV irradiation.
Supplementary Fig. 9 ^{13}C NMR spectra of CPI and PPI.
Supplementary Fig. 10 The coarse-grained model and process in the MD simulations.
Supplementary Fig. 11 UV-enhanced gelation kinetics of PPI solution with different HEMA grafting ratios.
Supplementary Fig. 12 The spinnability of PPI spinning solution.
Supplementary Fig. 13 The gelation depth of the CPI gel fibers.
Supplementary Fig. 14 The mechanical performance of CPI gel fibers with different HEMA grafting ratios.
Supplementary Fig. 15 Optical microscopy images of CPI gel fibers before and after solvent exchange.
Supplementary Fig. 16 The water contact angle of CPI aerogel fibers.
Supplementary Fig. 17 Cross-sectional SEM images of PPI-100 fiber
Supplementary Fig. 18 The N_2 sorption isotherms and pore size distribution of CPI aerogel fibers.
Supplementary Fig. 19 Shrinkage and density of CPI aerogel fibers.
Supplementary Fig. 20 The mechanical performance of CPI aerogel fibers.
Supplementary Fig. 21 Photograph of the CPI-100 aerogel fabric.
Supplementary Fig. 22 Thermal insulating performance of CPI aerogel fabric, commercial PI fabric and cotton.
Supplementary Fig. 23 Photograph and infrared thermal images of thermal puppets wearing CPI aerogel fabric and cotton.
Supplementary Fig. 24 Schematic of ITA textile and its structure change during heating.
Supplementary Fig. 25 Structure and physical properties of CPI/PCM fibers.
Supplementary Fig. 26 Shape memory of CPI/PCM fiber.
Supplementary Fig. 27 Shape memory of CPI/PCM fabric.
Supplementary Fig. 28 Intelligent thermoregulating performance of ITA textile.
Supplementary Fig. 29 Shape fixity and recovery ratios of ITA textile.
Supplementary Fig. 30 Temperature-time curves of ITA textile and cotton fabric.
Supplementary Fig. 31 The cyclic stability of the CPI/PCM fabric.

Supplementary Table 1. Solubility of PI.
Supplementary Table 2. Formulation of synthetic PPI.
Supplementary Table 3. Solubility of PPI-100.
Supplementary Table 4. Time consumption of preparing aerogel fibers through four gelation strategies reported previously.
Supplementary Table 5. Summary of temperature difference/thickness (|ΔT|/T) values for commercial materials and reported aerogel fibers.

Supplementary references

1. Coarse-grained molecular dynamics simulations.

A coarse-grained (CG) molecular dynamics (MD) model of photosensitive polyimide molecules was constructed, as illustrated in Supplementary Fig. 10. In the present CG MD model, a bead represents a cluster of atoms, involving plenty of repeat units. The polyimide molecule (Supplementary Fig. 10a) is modelled by a linear beads-spring chain consisting of L_P beads linked by L_P-1 bonds (Supplementary Fig. 10b), where that L_P is fixed at 23 without changing in the present work. Each polyimide molecule possesses m functionally reactive beads (red beads in Supplementary Fig. 10b) uniformly spaced along the polyimide molecular chain, corresponding to the cross-linkable double bond groups grafted to the polyimide backbone in the experiment. The content of cross-linkable groups (denoted by f) can be calculated by

$$f = m/L_P \quad (1)$$

The solvent molecule is modelled by a single bead (denoted by S). The mass and diameter of each bead (including polyimide and solvent) is set to be m and σ. As shown in Supplementary Fig. 10c, when the reactive beads meet each other within the reaction

radius R ($R = 1.0\sigma$), the two nearest cross-linkable beads have the chance to react with each other at a certain probability P_r to form a new (covalent) bond. The reaction probability P_r can also be used as a parameter that controls the reactivity determined by the nature of the reactive groups. In this work, the reaction probability P_r is set to be 0.001.

The MD simulations include both bonding U_{bond} and nonbonding potentials U_{ij} in the interaction potential. Any pair of ith and jth beads has a nonbonding potential U_{ij} which can be calculated using the modified Lennard-Jones 12:6 (LJ126) potential.

$$U_{ij} = \begin{cases} 4\varepsilon_{ij}\left[\left(\frac{\sigma}{r_{ij}}\right)^{12} - \left(\frac{\sigma}{r_{ij}}\right)^{6} - \left(\frac{\sigma}{r_{ij}{}^{c}}\right)^{12} + \left(\frac{\sigma}{r_{ij}{}^{c}}\right)^{6}\right], & r_{ij} \le r_{ij}{}^{c} \\ 0, & r_{ij} > r_{ij}{}^{c} \end{cases} \quad (2)$$

where the ε_{ij} is the interaction parameter between beads i and j. When r_{ij} is truncated and shifted to zero energy and force, this distance is known as r_{ij}^{c}. In the modified LJ126 potential, the cutoff distance (r_{ij}^{c}) determines the attractive ($r_{ij}^{c} > 2^{1/6}\sigma$) or repulsive ($r_{ij}^{c} \le 2^{1/6}\sigma$) interaction between i and j beads, where the σ represents the distance unit in the MD simulations. To mimic the corresponding experimental system, the strength and cutoff distance for the interactions between beads i and j is fixed at 1.0ε and 2.5σ (attractive), respectively, where the ε represents the unit of energy in the MD simulations.

Modified finite extensible nonlinear elastic (FENE) potential gives U_{bond}.

$$U_{\mathrm{bond}} = -0.5k_{\mathrm{b}}R_0{}^2 ln\left[1 - \left(\frac{r}{R_0}\right)^2\right] \quad (3)$$

where $k_b = 20\varepsilon/\sigma^2$ and $R_0 = 1.5\sigma$ is the elastic coefficient and the maximum extensible bond length, respectively. We used a cosine harmonic function (angle potential) to further constrain the linearly rigid chain structure of polyimide, written as

$$U_{\mathrm{angle}}(\theta) = \frac{1}{2}k_{\mathrm{a}}(cos\theta - cos\theta_0)^2 \quad (4)$$

where $k_a = 20\varepsilon$ is the angle spring constant and $\theta_0 = 180°$ is the equilibrium angle.

In the present MD simulation, the total number of CG MD beads is 48000, containing 834 polyimide molecules (each polyimide molecule chain includes $L_P = 23$ beads, i.e., the mass fraction of polyimide is $834L_P / 48000 \times 100\% \approx 40\%$). All the MD simulations were carried out by the large scale atomic/molecular massively parallel simulator (LAMMPS), developed by Sandia National Laboratories[1]. In the MD simulations, to generate the initial configurations, we constructed a large system with low volume fraction in a cubic box, which was compressed to the volume fraction of 0.45. Based on the initial configurations, the MD simulations were performed in the isothermal-isobaric (NPT) ensemble by using the Nose-Hoover barostat and thermostat. During the MD simulations, the periodic boundary conditions were imposed with a time step $\Delta t = 0.001\tau$ (τ denotes the unit time).

2 Supplementary Figures and Tables

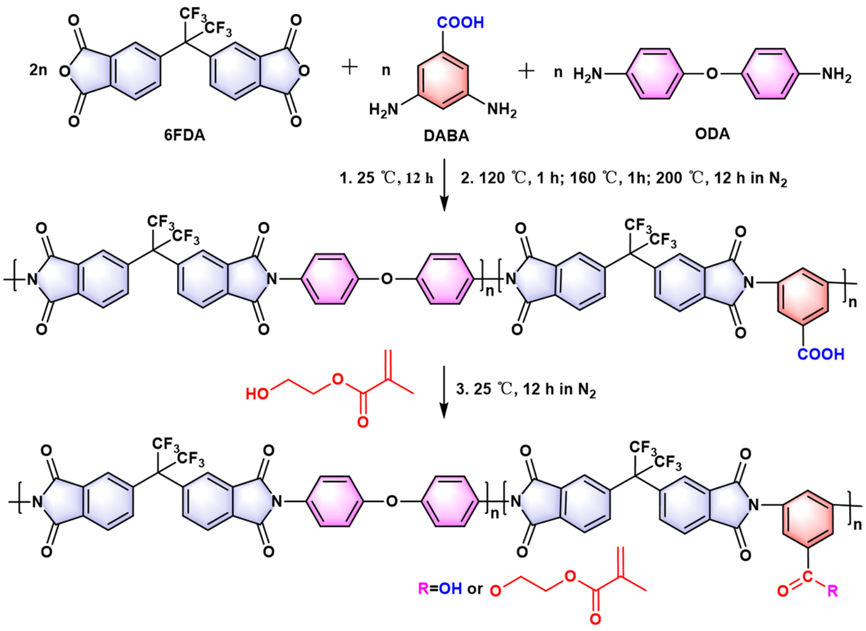

Supplementary Fig. 1 Synthetic chemical reaction of photosensitive polyimide (PPI).

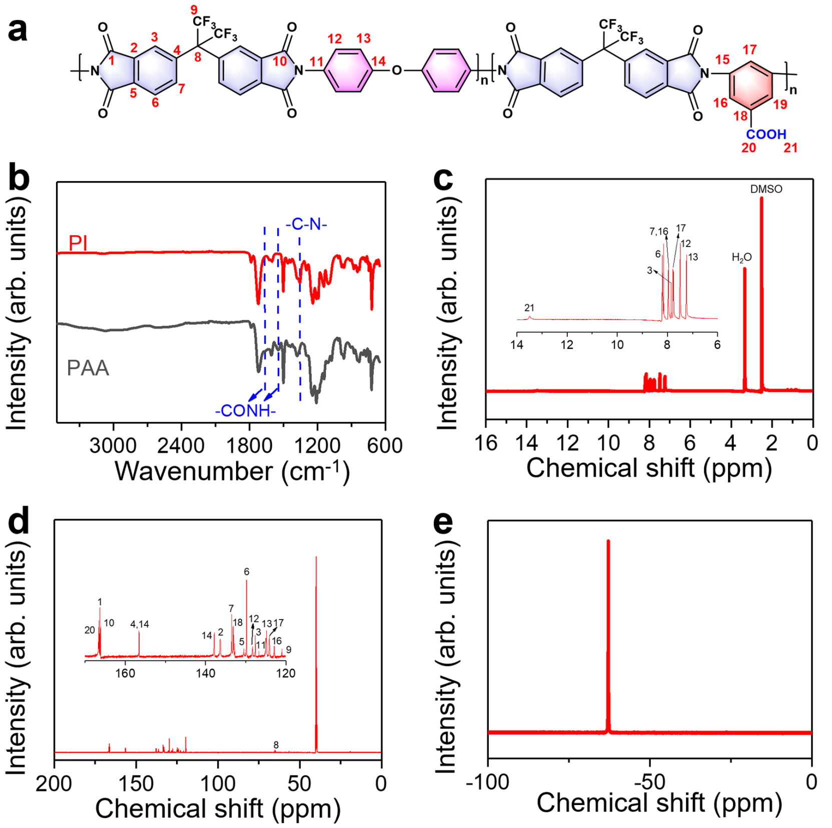

Supplementary Fig. 2 (a) Chemical structure of PI. (b) FTIR spectra of PI and PAA.

(c) ^{1}H NMR spectrum of PI. (d) ^{13}C NMR spectrum of PI. (e) ^{19}F NMR spectrum of PI.

Structure of the PI was determined by ^{1}H NMR, ^{13}C NMR, ^{19}F NMR and FT-IR spectra. The FT-IR spectra of PI showed two peaks assigned to the carbonyl group, 1355 cm^{-1} (C-N stretching) and 1784 cm^{-1} (C=O asymmetric stretching), which represented the characteristic peaks of the polyimides. The absorption peak near 1145 cm^{-1} was assigned to the C-F bond, while the strong peaks appearing at 1100 cm^{-1} is assigned to the ether bond vibration peaks. The peaks at 1498 cm^{-1} and 1451 cm^{-1} were assigned to the C-C stretching of the aromatic rings[2]. The ^{1}H NMR spectrum of PI showed the main chain proton signals for aromatics at 7.2-8.25 ppm. Characteristic signals of the carboxyl group (166.6 ppm), the imide ring (166.3 and 166.1 ppm), the C-F and the benzene ring (140-120 ppm) are clearly observed in the ^{13}C NMR pattern. And in ^{19}F NMR spectrum, the characteristic the signal of -CF_3 appeared at -63.63 ppm[3]. The successful synthesis of the expected PI was clearly indicated by the NMR and FTIR spectra.

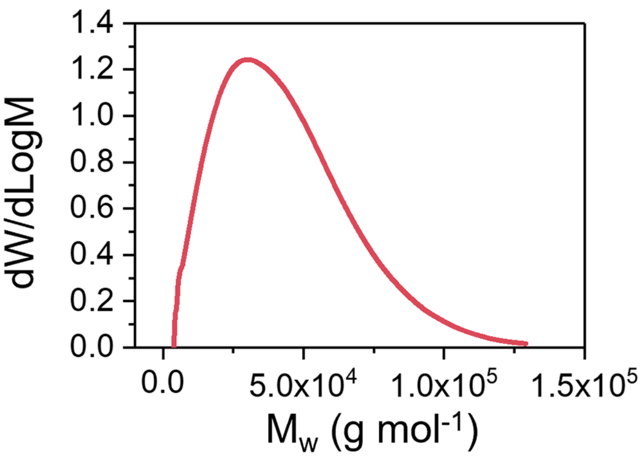

Supplementary Fig. 3 Molecular weight distribution curve of soluble PI.

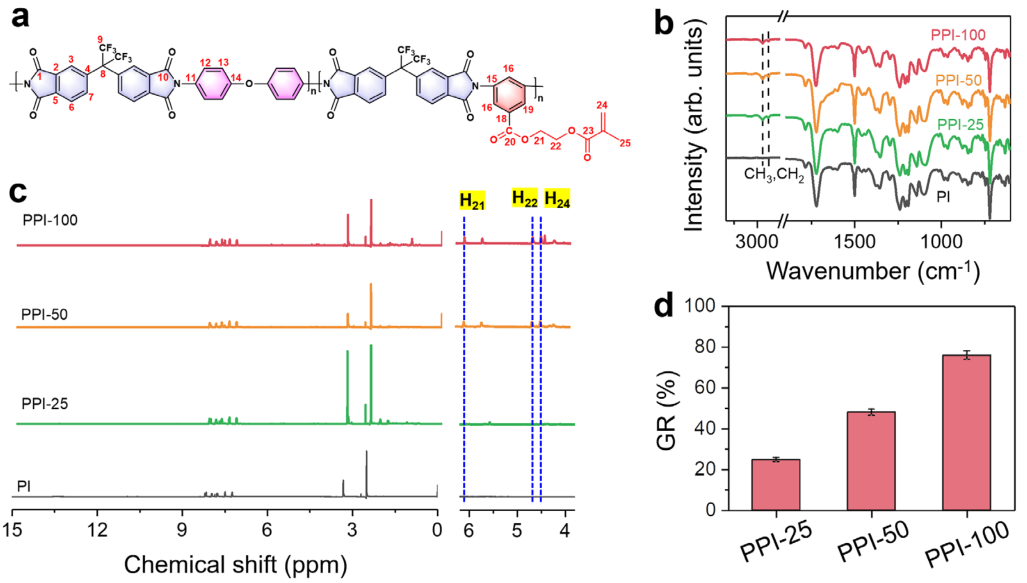

Supplementary Fig. 4 a) Chemical structure of PPI-100. b) FTIR spectra of PPI with different HEMA grafting ratios. c) ^{1}H NMR spectra of PPI with different HEMA grafting ratios. d) Practical grafting ratio (GR) of PPI solutions with different HEMA grafting ratios. Error bars represent the standard deviation.

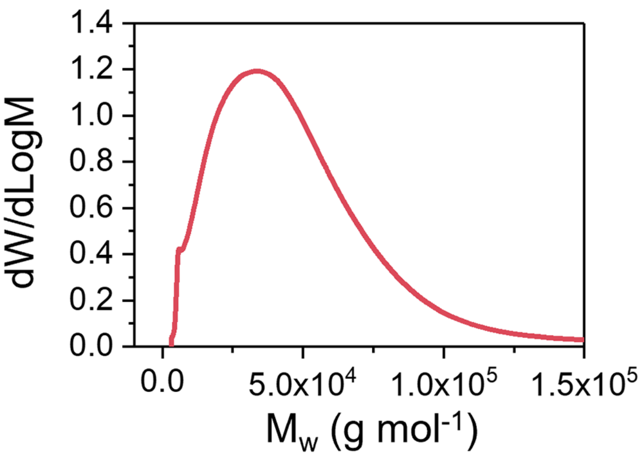

Supplementary Fig. 5 Molecular weight distribution curve of PPI.

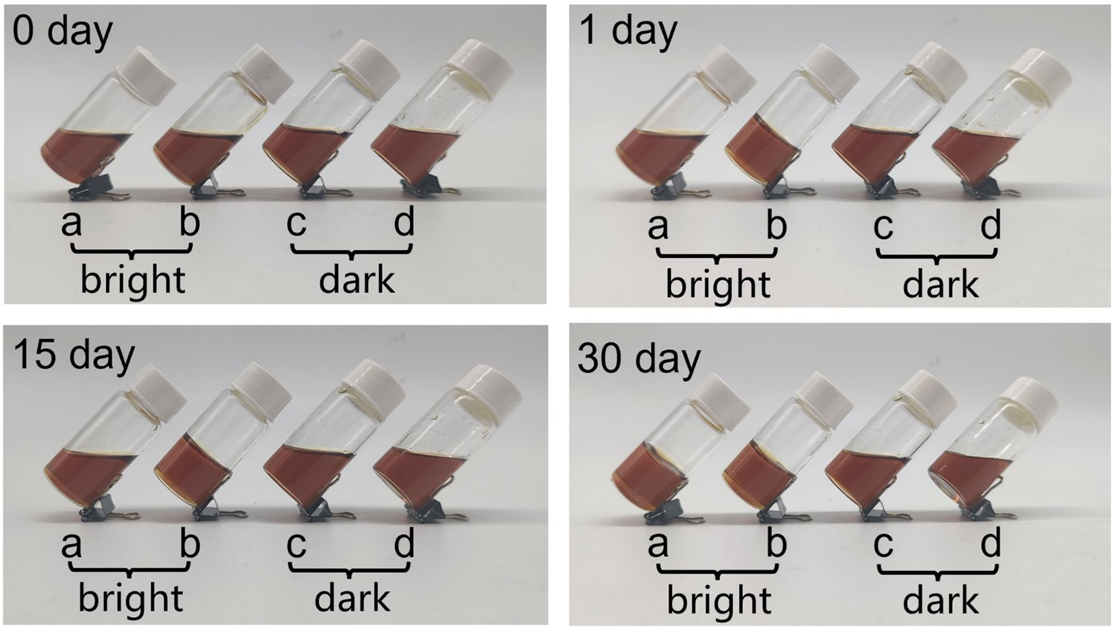

Supplementary Fig. 6 Storage stability experiments of PPI spinning solution. a represents PPI solution without photoinitiator (Irgacure2100). b represents PPI solution with photoinitiator (Irgacure2100). c represents PPI solution without photoinitiator (Irgacure2100) stored away from light. d represents PPI solution with photoinitiator (Irgacure2100) stored away from light.

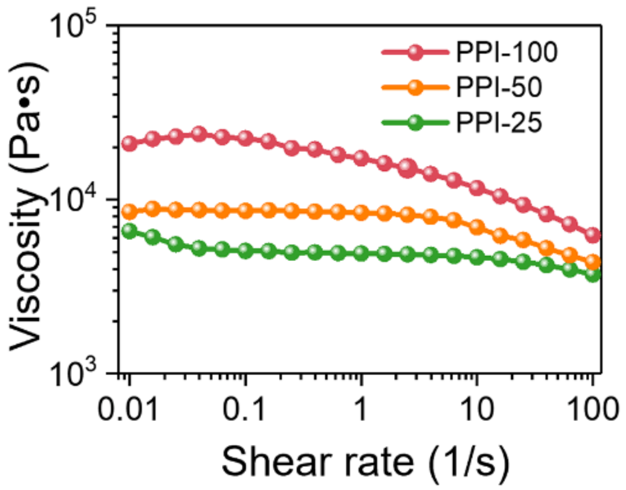

Supplementary Fig. 7 Apparent viscosity as a function of shear rate for PPI solution with different HEMA grafting ratios.

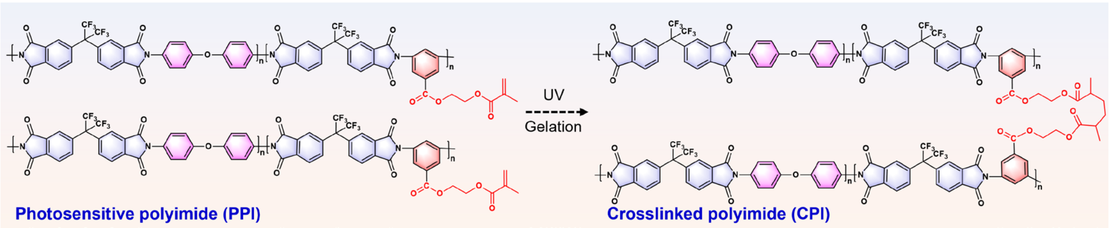

Supplementary Fig. 8 Chemical structure of PPI and CPI under UV irradiation.

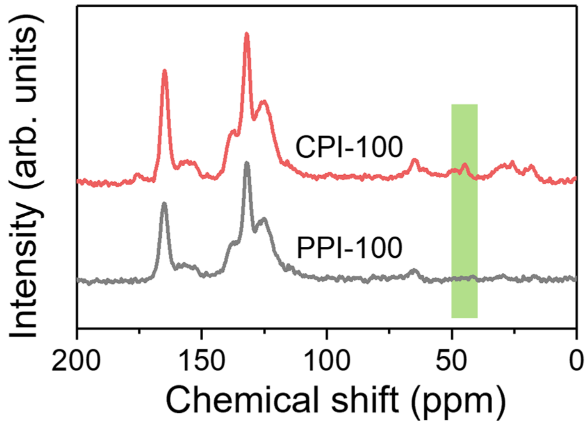

Supplementary Fig. 9 ^{13}C NMR spectra of CPI-100 and PPI-100.

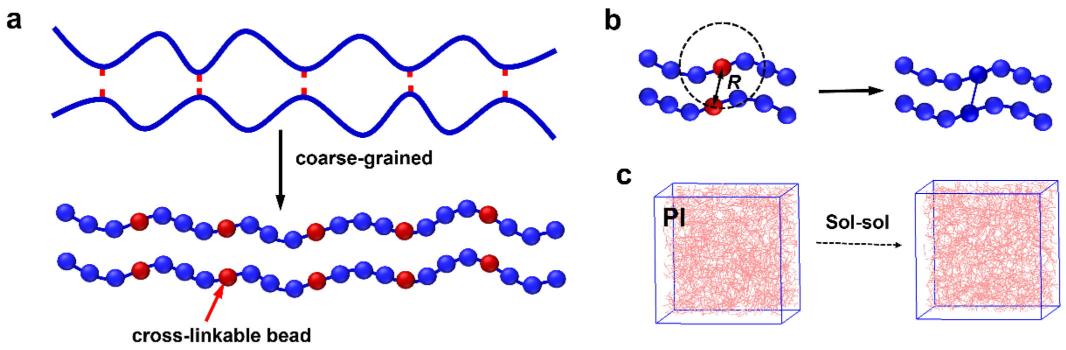

Supplementary Fig. 10 a) Sketch of photosensitive polyimide molecules and corresponding coarse-grained model of photosensitive polyimide molecules. b) The reaction process in the MD simulations. c) Coarse-grained molecular dynamics simulation of sol-gel transition of PI (non-crosslinkable) solution.

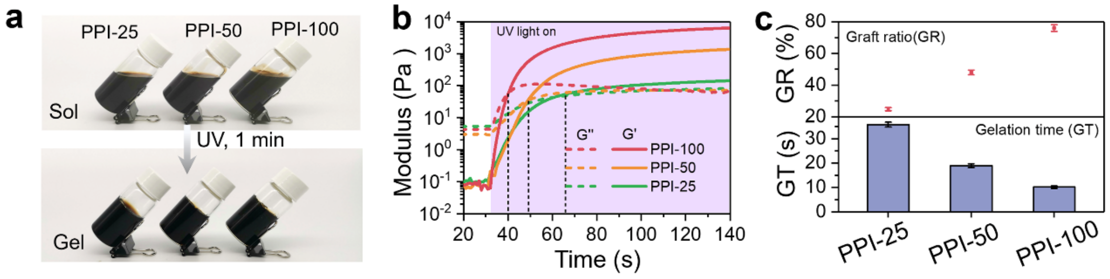

Supplementary Fig. 11 a) Photographs showing UV-enhanced gelation of PPI solution with different HEMA grafting ratios. The PPI with different grafting ratios formed stable gels after UV irradiation for 1 min. b) UV-enhanced gelation kinetics of PPI solution with different HEMA grafting ratios. The gelation point of PPI-25 and PPI-50 are 36 s and 19 s, respectively. c) Practical grafting ratio (GR) and gelation time (GT) of PPI solutions with different HEMA grafting ratios. Calculated by ^{1}H NMR spectroscopy, the graft ratio of PPI-25, PPI-50 and PPI-100 are 24.8%, 47.9%, and 76%, respectively. Therefore, the gelation time decreases with increasing grafting ratio, due to that the high grafting ratio provides more reaction sites. Error bars represent the standard deviation.

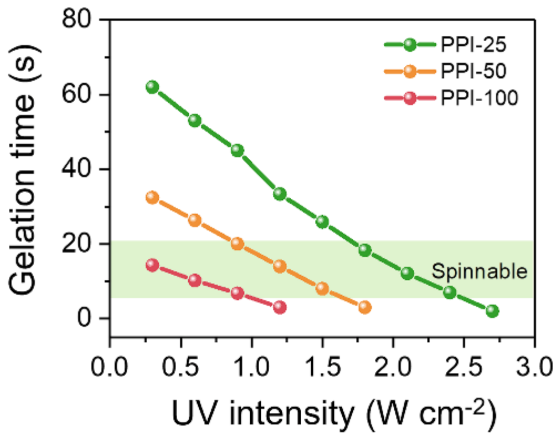

Supplementary Fig. 12 The gelation time as a function of UV intensity for PPI solution with different HEMA grafting ratios.

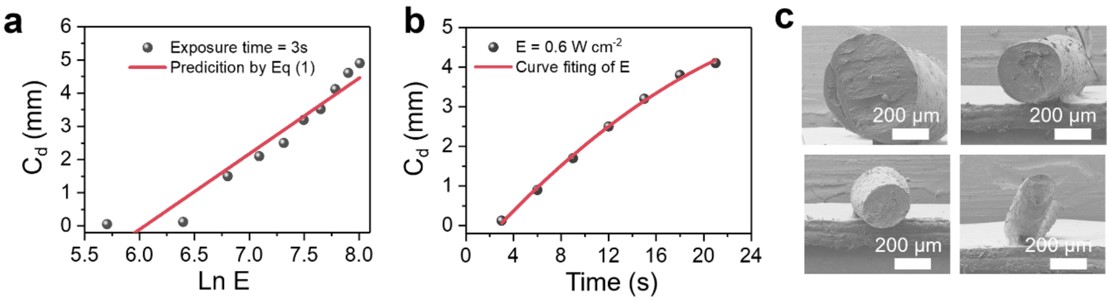

Supplementary Fig. 13 a) The gelation depth of CPI-100 gel fiber as a function of UV intensity when the exposure time was 3 s. b) The gelation depth of CPI-100 gel fiber as a function of exposure time when the exposure energy was 0.6 W cm^{-2}. c) SEM images of CPI-100 aerogel fibers with different diameters.

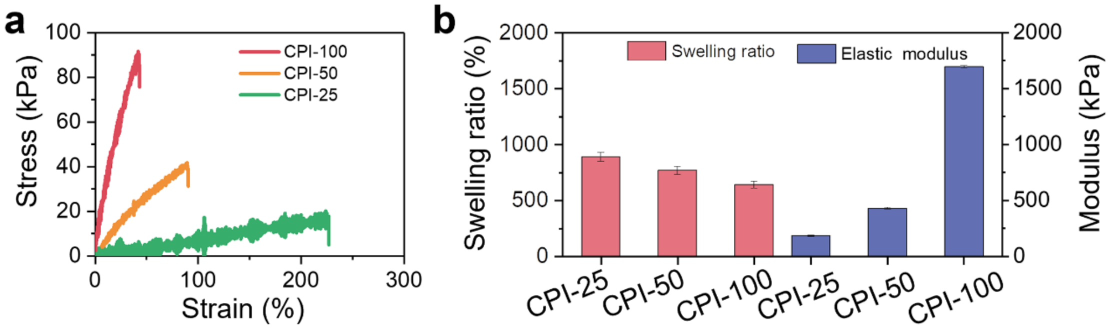

Supplementary Fig. 14 a) Stress-strain curves of CPI gel fiber with different HEMA grafting ratios. b) Swelling ratio and modulus of CPI gel fiber with different HEMA grafting ratios. The tensile strength increases from 16 kPa to 90 kPa when the HEMA grafting ratio increases from 25 to 100. This is attributed to the higher crosslinking density (low swelling ratio = 185%) and elastic modulus (1696 kPa) of CPI-100. Error bars represent the standard deviation.

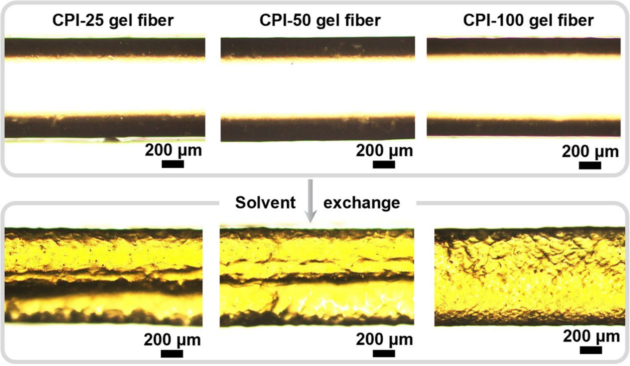

Supplementary Fig. 15 Optical microscopy images of CPI gel fiber with different grafting ratios before and after solvent exchange. After solvent exchange, the structure of CPI-25 and CPI-50 gel fibers is deformed and grooves appear on the fiber surface. When increasing the crosslinking density of CPI, the surface grooves of CPI-100 gel fibers disappear.

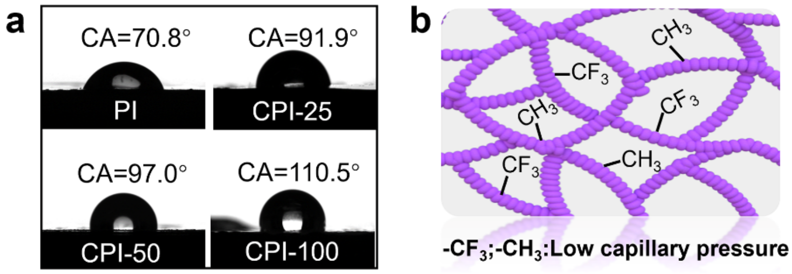

Supplementary Fig. 16 a) The water contact angle (CA) of CPI aerogel fibers with different HEMA grafting ratios. The CA increases from 70.8° to 110.5° when the HEMA grafting ratio increases from 0 to 100. This is mainly attributed to the following reasons: (1) PPI grafted with HEMA consumes the hydrophilic carboxyl group on PPI, (2) the hydrophobic methyl group on HEMA can reduce the surface energy. b) Mechanism of CPI aerogel fiber under ambient pressure drying. The CPI-100 with abundant methyl/trifluoromethyl groups exhibits hydrophobic surface and low capillary

pressure, which can further avoid structural collapse during ambient pressure drying.

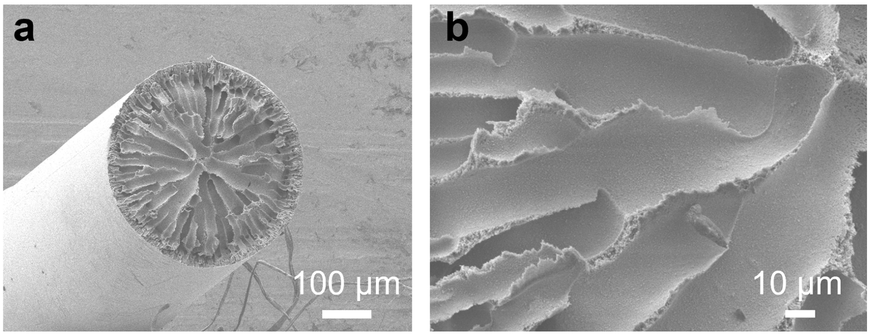

Supplementary Fig. 17 Cross-sectional SEM images of PPI-100 fiber at a) low and b) high magnifications. The PPI-100 fibers were prepared by squeezing the PPI-100 spinning solution into coagulation bath (ethanol) and leaving it for 30 min, followed by successive ethanol washes and ambient pressure drying.

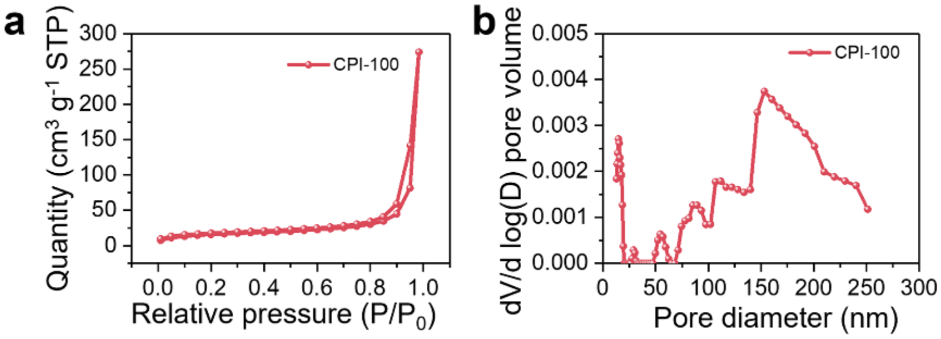

Supplementary Fig. 18 a) N_2 sorption isotherms at 77 K of CPI-100 aerogel fiber. The sorption curves show typical type IV isotherm characteristics with an adsorption hysteresis, indicating the presence of mesopores. b) Pore size distribution of CPI-100 aerogel fiber derived from Barrett–Joyner–Halenda (BJH) analysis (*V*, pore volume; *w*, pore width).

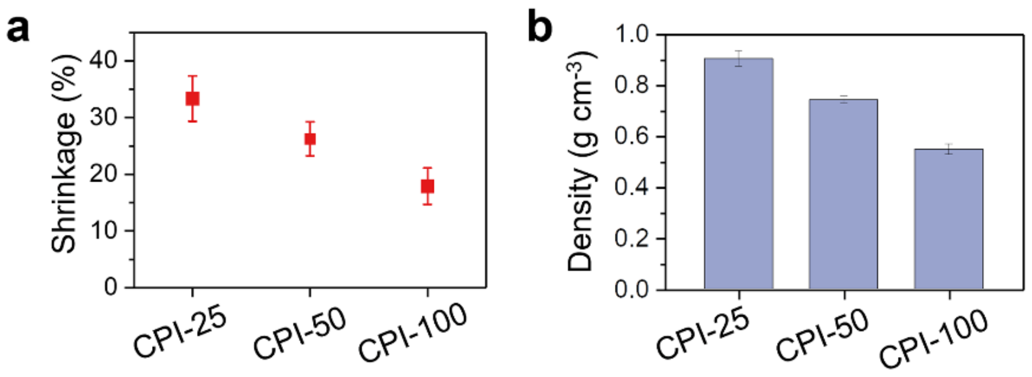

Supplementary Fig. 19 a) Shrinkage and b) density of CPI aerogel fibers with different HEMA grafting ratios. Error bars represent the standard deviation.

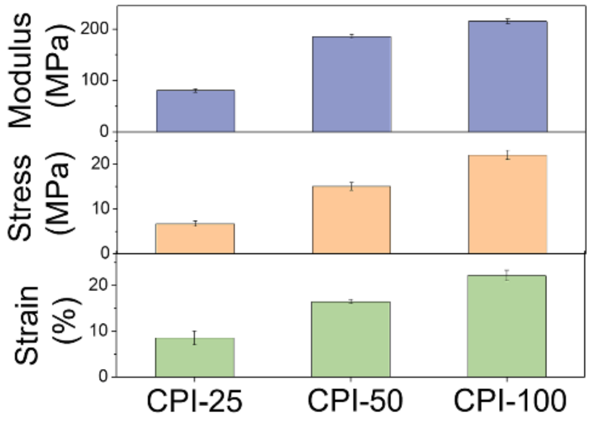

Supplementary Fig. 20 Tensile strain, tensile stress and tensile modulus of CPI aerogel fibers with different grafting ratios. Error bars represent the standard deviation.

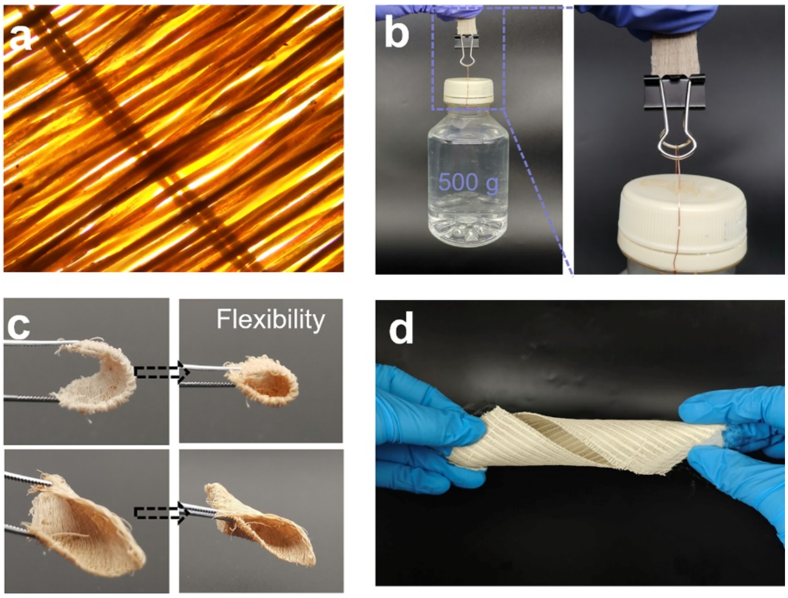

Supplementary Fig. 21 a) Optical microscopy images of CPI aerogel fabric. b) Photograph shows that the structure of the CPI-100 aerogel fabric remains intact when loaded with a weight of 500 g. c-d) Photographs showing the flexibility of CPI aerogel fabric in different bending directions.

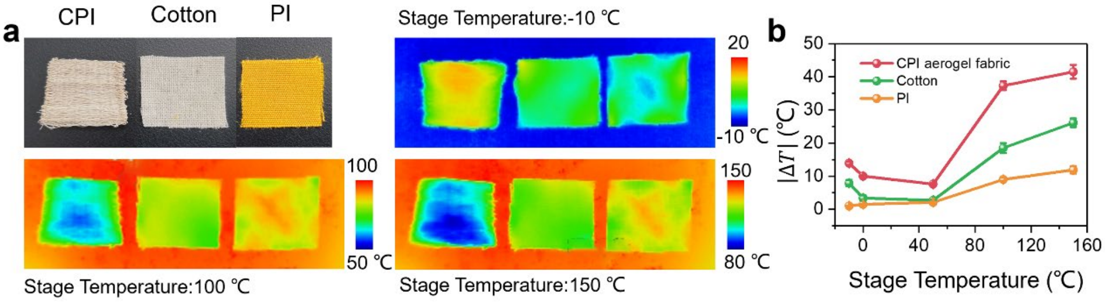

Supplementary Fig. 22 a) Photographic and infrared thermal images of CPI aerogel fabric, commercial PI fabric and cotton on the hot and cool stages. b) Temperature difference (|ΔT|) between the fabric surface and the hot/cool stage plotted against the stage temperature for all the fabrics. For the as-prepared CPI aerogel fabric, and two other commercial fabrics (i.e., PI and cotton fabrics), the CPI aerogel fabric showed a maximum absolute temperature difference (|ΔT|) of 13.9 °C at -10 and 41.5 °C at 150 °C,

which was significantly higher than those of the other two fabrics. Error bars represent the standard deviation.

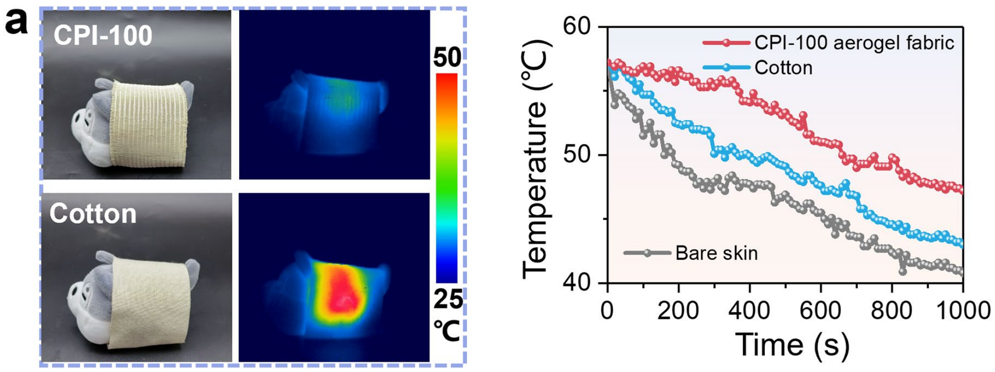

Supplementary Fig. 23 a) Photograph and infrared thermal images of thermal puppets wearing CPI aerogel fabric and cotton, and b) corresponding surface temperature of the thermal puppets recorded by a thermal couple. Thermocouples recorded the temperature change of the bare thermal puppet and that wearing CPI-100 aerogel fabric and cotton fabric (Fig. 4f). For the bare thermal puppet, the temperature drops rapidly from 58.0 °C to 49.0 °C after 300 s. For the thermal puppet covered with CPI-100 aerogel fabric, the temperature remains at 56.4 °C after 300 s, indicating a good heat preservation performance of CPI-100 aerogel fabric.

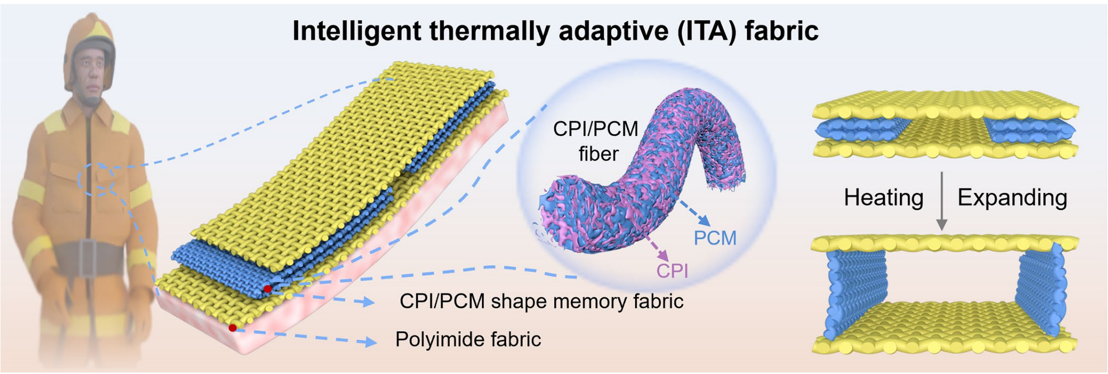

Supplementary Fig. 24 Schematic of ITA textile and its structure change during

heating.

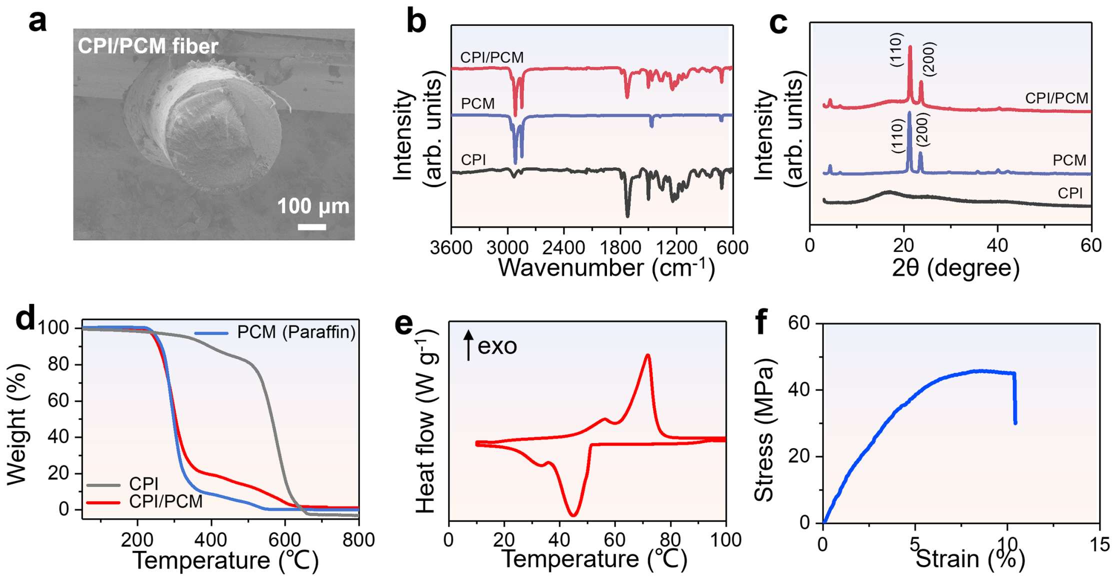

Supplementary Fig. 25 a) Cross-sectional morphology of CPI/PCM fiber. b) FTIR spectra of CPI, PCM and CPI/PCM. From the FTIR spectrum of PCM (paraffin), C–H asymmetric stretching vibration and symmetric vibration can be observed at 2915 cm^{-1} and 2850 cm^{-1}, respectively. The C–H bending vibration occurs in the characteristic peaks at 1460 cm^{-1} and 1376 cm^{-1}. The CH_2 rocking vibration of PCM is found at 727 cm^{-1}. In the CPI, the characteristic band that appeared at 1778 cm^{-1} and 1725 cm^{-1} were the imide absorption peaks[4]. From the comparative analysis of the FTIR spectra, the characteristic peaks of paraffin appeared in CPI/PCM, indicating that successful composite of PCM with CPI aerogel fiber. c) X-ray diffraction (XRD) patterns of CPI, PCM and CPI/PCM. Two sharp diffraction peaks at 21.4° and 23.7° were observed for pure paraffin, which corresponded to the (110) and (200) diffraction planes of paraffin crystal[5], respectively. The XRD patterns of CPI/PCM showed almost the same diffraction peaks as paraffin at 21.4° and 23.7°. d) Thermogravimetric analysis (TGA) curves of CPI, PCM and CPI/PCM. e) DSC curves of CPI/PCM. f) Tensile stress-strain

curve of CPI/PCM fiber.

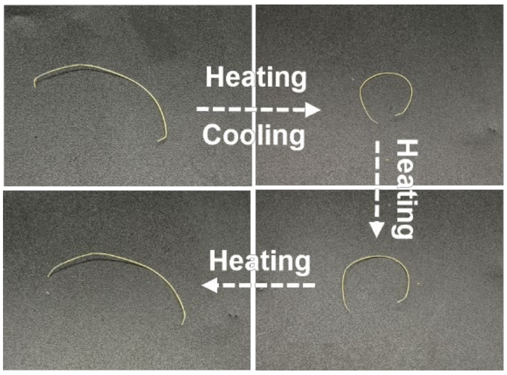

Supplementary Fig. 26 Experimental demonstration of shape memory of CPI/PCM fiber. The CPI/PCM fabrics are programmed by heating to 80 °C and fixed by cooling to 20 °C.

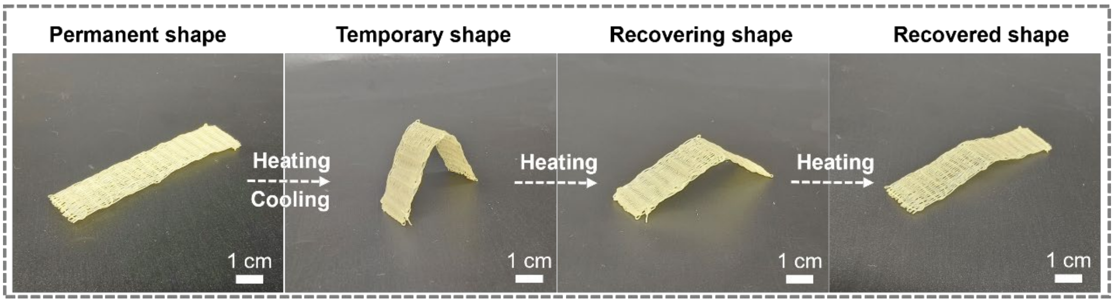

Supplementary Fig. 27 Experimental demonstration of shape memory of CPI/PCM shape memory fabric.

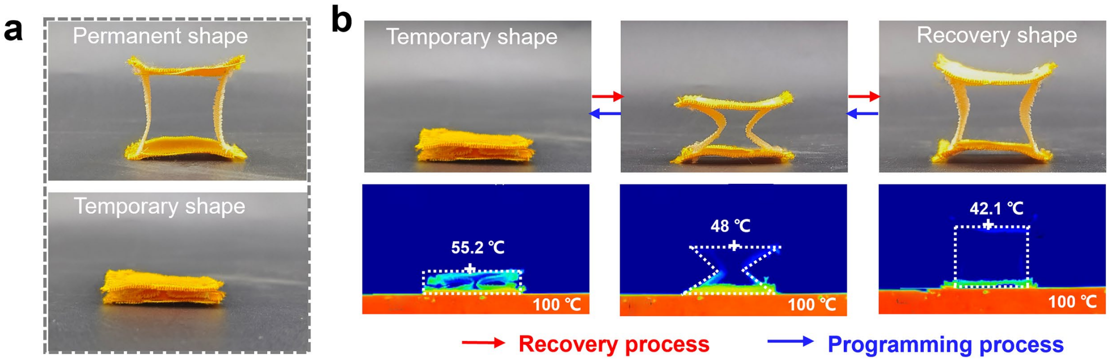

Supplementary Fig. 28 a) Photography of ITA textile in permanent shape and temporary shape. b) Optical and infrared thermal images of ITA textile during deformation process on a hot stage with 100 °C.

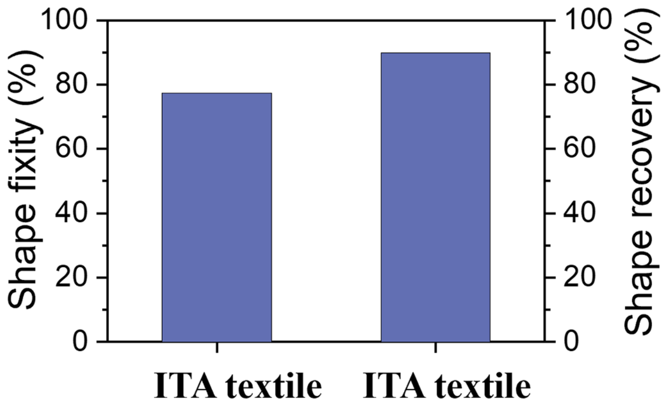

Supplementary Fig. 29 Shape fixity and recovery ratios of ITA textile.

The shape fixity ratio (R_f) and shape recovery ratio (R_r) were calculated using eqn (1) and (2).

$$R_f = \frac{H_{unload} - H_0}{H_{load} - H_0} \times 100\% \quad (5)$$

$$R_r = \frac{H_{unload} - H_{rec}}{H_{unload} - H_0} \times 100\% \quad (6)$$

Where H is the height of ITA textile.

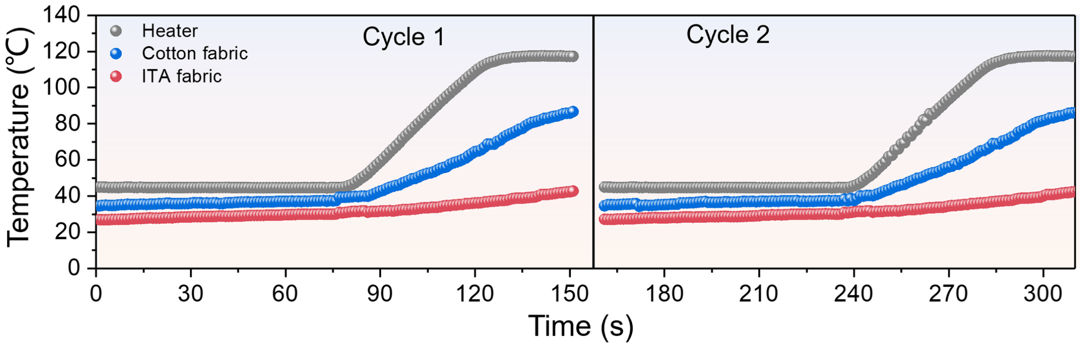

Supplementary Fig. 30 Temperature-time curves of ITA textile, cotton fabric and heater from 45 °C to 120 °C in different cycles. When the temperature of the stage increases from 45.0 °C to 120.0 °C, the surface temperature of the cotton fabric sharply increases to 87 °C, while ITA textile still maintains a low surface temperature of 42.1 °C.

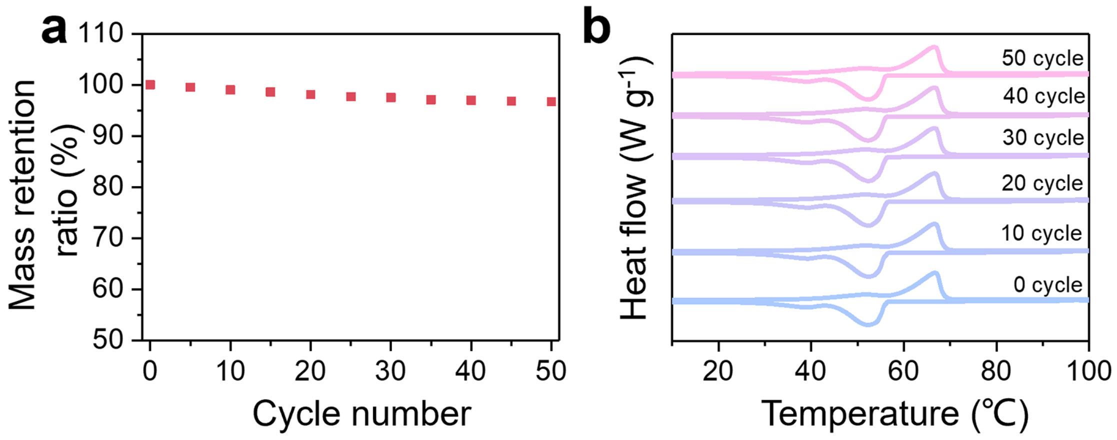

Supplementary Fig. 31 (a) The mass retention ratio of the CPI/PCM fabric after 50 cycles of heating and cooling. (b) DSC curves of CPI/PCM fabric after different heating and (b) cooling cycle. The mass retention ratio of the CPI/PCM fabric after 50 heating-cooling cycles was 96.7%. The DSC curves after 50 heating-cooling cycles are almost coincident with that of the original one, reflecting the cyclic stability of PI/PCM fabric. This is mainly contributed to the fact that the CPI aerogel fibers with a high specific surface area and abundant mesopores would have strong capillary force to confine phase-change materials from leakage.

Supplementary Table 1 Solubility of PI.

Sample	Solvent							
	**NMP**	**DMAc**	**DMF**	**DMSO**	**Acetone**	**CH_2Cl_2**	**$CHCl_3$**	**THF**
**PI**	++	++	++	++	++	+-	+-	++

++: soluble at room temperature, +-: partially swelling at room temperature.

Supplementary Table 2 Formulation of synthetic PPI.

Sample	ODA/g	DABA/g	6FDA/g	HEMA/g	DCC/g	DMAP/g
**PI**	1.001	0.7608	4.4423	/	/	/
**PPI-25**	1.001	0.7608	4.4423	0.1625	0.2507	0.0152
**PPI-50**	1.001	0.7608	4.4423	0.325	0.5015	0.0304
**PPI-100**	1.001	0.7608	4.4423	0.65	1.03	0.0608

Supplementary Table 3 Solubility of PPI-100.

**Sample**	**Solvent**							
	**NMP**	**DMAc**	**DMF**	**DMSO**	**Acetone**	**CH_2Cl_2**	**$CHCl_3$**	**THF**
**PPI-100**	++	++	++	++	++	++	++	++

++: soluble at room temperature.

Supplementary Table 4 Time consumption of preparing aerogel fibers through four gelation strategies reported previously.

Aerogel	Strategy	Gelation/ Aging time	Solvent exchange	Drying method	Drying time	Refs
Silica aerogel fiber	Condensation reaction	5~15 s/ 3 h	/	Supercritical drying	/	6
Silica aerogel fiber	Condensation reaction	72 h	/	Vacuum drying	24~48 h	7
Silica aerogel	Condensation reaction	4	/	Ambient pressure drying	72 h	8
Silica aerogel	Condensation reaction	/	96 h	Ambient pressure drying	42 h	9
Polyimide aerogel fiber	Chemical crosslinking	30 min/48 h	36 h	Supercritical drying	10 h	10
Graphene aerogel fiber	Ionic crosslinking	5 h/24~72 h	48 h	Supercritical drying	12 h	11
MXene aerogel fiber	Ionic crosslinking	2 h	48 h	Supercritical drying	12 h	12
**CPI aerogel fiber**	**UV-enhanced gelation**	**10 s**	**2 h**	**Ambient pressure drying**	**5 h**	**This work**

Supplementary Table 5 Summary of temperature difference/thickness (|ΔT|/T) values for commercial materials and reported aerogel fibers.

**Sample**		**Temperature difference/|ΔT| (℃)**	**Thickness/T (mm)**	**|ΔT|/T (℃ mm^{-1})**	**Refs**
**Commercial materials**	PI	110	6.9	15.9	/
	Cotton	110	6.5	16.9	/
	Down	110	5.4	20.4	/
**Aerogel fiber**	PI aerogel fiber	50	1	50	13
	PI/MXene aerogel fiber	40	0.7	53	14
	Silica/BC aerogel fiber	80	1.4	57	15
	Aramid aerogel fiber	106	1.45	73.1	16
	PI aerogel fiber	110	1.1	100	17
**CPI aerogel fiber**		**110**	**0.7**	**157**	**This work**

Supplementary references:

1. Plimpton S. Fast parallel algorithms for short-range molecular dynamics. *J. Comput. Phys.* **117**, 1-19 (1995).
2. Noda T., Iwasaki T., Takada K. & Kaneko T. Soluble biobased polyimides from diaminotruxinic acid with unique bending angles. *Macromolecules* **54**, 10271-10278 (2021).
3. Jia M., Li Y., He C. & Huang X. Soluble perfluorocyclobutyl aryl ether-based polyimide for high-performance dielectric material. *ACS Appl. Mater. Interfaces* **8**, 26352-26358 (2016).
4. Feng J., et al. Aligned channel Gelatin@nanoGraphite aerogel supported form-stable phase change materials for solar-thermal energy conversion and storage. *Carbon* **201**, 756-764 (2023).
5. Bao Y., Lyu J., Liu Z., Ding Y. & Zhang X. Bending Stiffness-Directed Fabricating of Kevlar Aerogel-Confined Organic Phase-Change Fibers. *ACS Nano* **15**, 15180-15190 (2021).
6. Du Y., et al. Reaction-spun transparent silica aerogel fibers. *ACS Nano* **14**, 11919-11928 (2020).
7. Meng S., Zhang J.Y., Chen W.P., Wang X.P. & Zhu M.F. Construction of continuous hollow silica aerogel fibers with hierarchical pores and excellent adsorption performance. *Micropor. Mesopor. Mater.* **273**, 294-296 (2019).
8. Chen D., Wang X.D., Ding W.H., Zou W.B., Zhu Q. & Shen J. Silica aerogel monoliths derived from silica hydrosol with various surfactants. *Molecules* **23**,

3192 (2018).

9. Tang X.B., et al. A novel silica nanowire-silica composite aerogels dried at ambient pressure. *Mater. Des.* **115**, 415-421 (2017).

10. Li X., Dong G.Q., Liu Z.W. & Zhang X.T. Polyimide aerogel fibers with superior flame resistance, strength, hydrophobicity, and flexibility made via a universal sol-gel confined transition strategy. *ACS Nano* **15**, 4759-4768 (2021).

11. Hou Y.L., Sheng Z.Z., Fu C., Kong J. & Zhang X.T. Hygroscopic holey graphene aerogel fibers enable highly efficient moisture capture, heat allocation and microwave absorption. *Nat. Commun.* **13**, 1227 (2022).

12. Li Y.Z. & Zhang X.T. Electrically conductive, optically responsive, and highly orientated $Ti_3C_2T_x$ MXene aerogel fibers. *Adv. Funct. Mater.* **32**, 2107767 (2021).

13. Xue T.T., Zhu C.Y., Feng X.L., Wali Q., Fan W. & Liu T.X. Polyimide aerogel fibers with controllable porous microstructure for super-thermal insulation under extreme environments. *Adv. Fiber Mater.* **4**, 1118-1128 (2022).

14. Wang D., et al. Hierarchically porous polyimide aerogel fibers based on the confinement of $Ti_3C_2T_x$ flakes for thermal insulation and fire retardancy. *Compos. Commun.* **37**, 101429 (2023).

15. Sai H.Z., et al. Robust silica-bacterial cellulose composite aerogel fibers for thermal insulation textile. *Gels* **7**, 145 (2021).

16. Li M.M., Chen X., Li X.T., Dong J., Zhao X. & Zhang Q.H. Controllable strong and ultralight aramid nanofiber-based aerogel fibers for thermal insulation applications. *Adv. Fiber Mater.* **4**, 1267-1277 (2022).

17. Li M.M., Gan F., Dong J., Fang Y.T., Zhao X. & Zhang Q.H. Facile preparation of continuous and porous polyimide aerogel fibers for multifunctional applications. *ACS Appl. Mater. Interfaces* **13**, 10416-10427 (2021).
