# Supplementary material for: Fast and scalable production of crosslinked polyimide aerogel fibers for ultrathin thermoregulating clothes
Source: Nat Commun. 2023 Dec 16;14:8378. doi: 10.1038/s41467-023-43663-8 (PMC10725485; doi:10.1038/s41467-023-43663-8)
Supplement: Supplementary file 3 — Description of additional supplementary files [file 41467_2023_43663_MOESM3_ESM.pdf]

Description of additional supplementary files

Supplementary Movie 1: Wet-spinning via UV-enhanced dynamic gelation strategy.

Supplementary Movie 2: Extrusion of polyimide solution under UV irradiation.

Supplementary Movie 3: Extrusion of photosensitive polyimide solution under UV irradiation.

Supplementary Movie 4: Deformation process of intelligent thermal adaptive fabric.
